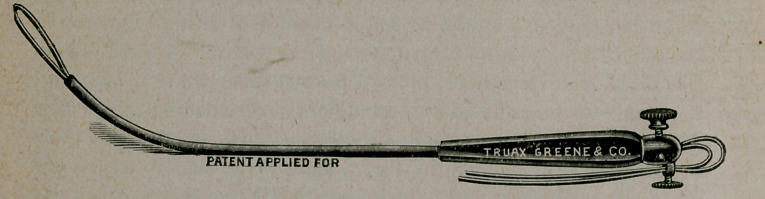# A New Method for the Management of Abortion

**Published:** 1892-05

**Authors:** Charles H. Harris

**Affiliations:** Cedartown, Ga.


### A NEW METHOD FOR THE MANAGEMENT OF ABORTION.

By CHARLES H. HARRIS, M. D., Cedartown, Ga.

The instrumental management of abortion has not kept abreast with other branches of operative surgery. True, there have been many improvements in instruments, in curettes, rapid dilators, etc., yet on the whole, we are about in the same condition of thirty years ago. To my mind, the reason for this is very evident. In our search for new and improved instruments to do different work, we have made no effort to improve the operation. This remains practically the same it was in the early days of gynecology. The operation of the older writers differs only in the mode and means of its doing from the modern. Its divisions are now, and were thirty years ago, in three stages, (1) dilating the os; (2) detatchment of placenta; (3) the extraction of the ovum. Improvements, if any, have been in the first stage. Graduated dilators doing rapid work have taken the place of uterine tents. As for curettes and forceps, they have ever been varied and numerous, and were as skillfully used formerly as now. Still, the operation is the same in its ancient division of three stages. Fortunately for the women, antiseptics have come to our aid and done much to prevent the dangerous sequelæ that follow in the wake of our rough handling.

In the domain of midwifery, as elsewhere, it is hard to improve on the best effort of nature. In her finest moods she does her work well and in such superb style as to put to shame the highest endeavors of art. If we would learn wisdom from her counsels, we must study and endeavor to imitate her example. In the typical abortion of a healthy woman there is no division of this work. The os does not dilate before detachment begins, nor is detachment complete before expulsion begins. The three processes begin and proceed simultaneously and are produced by contractions of the uterus. More than this, the three stages are completed at the same moment in the typical abortion. With the last detachment of placenta the os is at its maximum expansion and the ovum is expelled from the uterus. Each process begins and proceeds simultaneously with the others, and they all depend on a force from behind. This is the typical abortion in a healthy woman, and it is accomplished with such ease and so little disturbance as not to need a doctor. Women do not take their beds for them. Is there anything in the operation now in vogue that copies this splendid work of nature? I think not.

It is the purpose of this paper to announce to the profession a new operation for abortion, one that simulates as near as possible the work when done by the healthy woman. In view of the immense amount of labor expended on the subject of abortion by my superiors, who have the best opportunities for research and observation, this announcement will seem presumptuous, and I apply myself to the task with becoming diffidence; not however without the assurance of complete success. The operation I propose, not only simulates the typical abortion, but it also invokes the co-operative powers of the uterus in the process of dilatation, detachment and expulsion, and they all proceed *pari passu* with it.

Before entering into details, I will give the reader something of my experience in this line of work. Years ago—about twenty, I encountered a case which, for want of instruments at hand, I managed with a wire-loop. It was my design to detach the placenta with this improvised instrument, and thereby relieve the hemorrhage; then to tampon the vagina and leave the expulsion of the ovum to the natural powers of the uterus. On attempting to withdraw the wire it hung upon the ovum, which had become entrapped in the loop. There was but one way out of this dilemma—to pull upon the wire. With moderate traction the entire ovum leaped into the vagina. This to me, was a revelation and an object study which has engaged much of my leisure thoughts. In the journals I noticed a similar experience with other physicians, one enterprising doctor relieving a case with a bucket bail. Seeing what the wires could do, I have continued to use them ever since. Sometimes, however, they would fail me. If too slender they would bend and double up; if too stout they would not take the course I wished. This led me to devise an instrument which would give me control of assorted wires, round and flat, beyond my sight in the depths of the gravid uterus. On a careful study of my failures, I learned that for these cases we needed curettes that were adjustable *in utero*, of small size to pass a narrow os, and capable of being enlarged at the will of the operator after entering the womb. I learned furthermore, that I could not make a perfect toilet of the organ with the round wires, and had my instrument so constructed as to carry a stout watch spring, which by upward pressure would adjust itself to the uterine wall, and by rotary movement would scrape off the residual placenta. As the operation I propose brings on the use of this instrument, it is necessary that I give a brief description of it. (See cuts.)

The curette snare consists of handle and shaft with continuous tunnel for carrying wires. It resembles very much the urethral sound with handle. It has two thumb screws on the handle for fixing and adjusting wires. It is thirteen inches in length and so constructed as to carry an assortment of wires round and flat. When armed with wire the smallest loop measures three-eighths of an inch, and being flat may be made with little force to pass the os of any pregnant uterus. After entering the organ the operator at his will may project any sized loop he wishes for curetting and snaring purposes. The loop, after ensnaring the ovum gives a traction power of a hundred pounds, which is amply sufficient to either extract it or divide it. The wires are of spring steel and elastic, with sufficient resistance to plough through and break up an ovum without damaging the more resistant walls of the uterus. The wires are automatic in that they adjust themselves to the uterine wall in doing their work.

The operation which I will now detail, it must be understood, is intended to apply to the first three months of gestation before the development of ossification. When the structures of the fœtus become firm and tough this instrument and operation are not suitable.

*Operation.*—If the patient is at all nervous and not tolerant of pain, use chloroform in Sims' position. With the patient under the anesthetic the surgeon is confronted with a problem almost purely mechanical, the emptying of the uterus with the tools before him. Cases of accidental abortion require no previous dilatation as the os is soft and patulous. Should there be any difficulty, a few stout strokes of a dilator will remove it.

The smallest loop of snare No. 1 with round wire previously dipped in antiseptic solution is passed beyond the os internum. With the loop fairly in the uterus the operator, if an average one, should be master of the situation. Enlarge the loop half an inch and *tighten the thumb screw.* If not tightened the wire will slip backwards and forwards and failure result. Remember this: *Tighten the thumb screw* before working. The surgeon now has within the uterus a curette larger and better than the finger nail for detaching purposes. He should endeavor to do this work, if possible, without rupturing the membranes as they serve to guide the loop in finding the attachments of the placenta, which may be defined as with a probe. Having reached them and having the bearings of the uterus clearly in mind, the work of separation should be brief and thorough. Grasping the handle of the instrument firmly, he urges the loop forward to its work. It is made to move forwards, backwards and laterally with force where it meets with resistance. Churn the uterus in every direction. Sweep the staff of the curette around the globe of the ovum and make sure of its complete severance. Now enlarge the loop to half its capacity and *tighten the thumb screw.* To make "assurance doubly sure" renew the churning and scrape the uterus, as you would a mortar, with a thin spatula. In this ordeal, if the membranes are not ruptured with snare No. 2 with small watch spring loop, the work is easily done. There could not be devised a better instrument for rupturing the membrane. For certainty, safety and celerity it cannot be excelled. Having ruptured the membranes the work of snaring begins. Snare No. 1 is reintroduced and a large loop sprung in the uterus. *Tighten the thumb screw*, and keep the snare moving until it hangs on the ovum. Now pull steadily and with increasing force until the ovum is either extracted or divided. In the latter event, repeat the operation until it is chopped into fragments easy of extraction. Snare No. 2 with watch spring wire is used for rupturing the membranes and to follow the work of No. 1. It gives the finishing touches to the operation and completes the toilet of the organ. It is now passed into the uterus and its loop enlarged until it reaches the fundus. *Tighten the thumb screw* and with upward pressure cause the loop to rotate backwards and forwards until the residual placenta is scraped off its walls. Now, remove the detritus of the ovum with the snare, mop out the organ with a styptic antiseptic and wash the vagina with warm sterilized water.

This is the new operation. From the time the loop is first passed into the uterus its powers are aroused. When you pull on an ensnared ovum you invoke its best efforts at expulsion. Thus there is a coincidence of the processes of dilatation, detachment and expulsion, and they progress *pari passu* with the operation.

---

DR. BASS' CASE OF IDIOSYNCRASY.—In reply to Dr. Bass' communication in the last JOURNAL, we have received the following:

KOSCIUSKO, MISS., April 12, 1882.

EDITOR JOURNAL: Heart's action good; respiration rapid and irregular; diagnosis, *hysteria.* Find morbid cause and circumstances connected with first attack, and when next called instituted Emmett's treatment—rubber tube. This will prove effective. DRS. COLEMAN & SMYTHE.

## Figures and Tables

**Figure f1:**
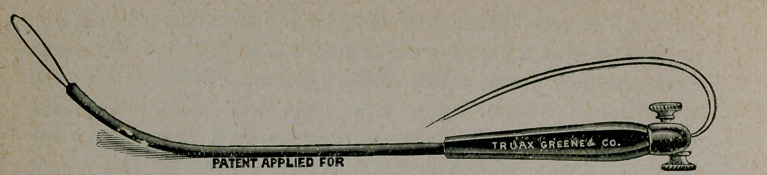


**Figure f2:**